# Percolation Network Formation in Nylon 6/Montmorillonite Nanocomposites: A Critical Structural Insight and the Impact on Solidification Process and Mechanical Behavior

**DOI:** 10.3390/polym14173672

**Published:** 2022-09-04

**Authors:** Tingzi Yan, Depei Chen, Baijin Zhao, Xiaodong Jiang, Lian Wang, Yongjin Li

**Affiliations:** 1 College of Material, Chemistry and Chemical Engineering, Key Laboratory of Organosilicon Chemistry and Material Technology, Ministry of Education, Hangzhou Normal University, Hangzhou 311121, China; 2 Jiangsu Boiln Plastics Company Limited, Zhangjiagang 215626, China

**Keywords:** nylon 6/montmorillonite nanocomposites, percolation network, solidification, toughness to brittleness transition

## Abstract

The incorporation of montmorillonite (MMT) into Nylon 6 can endow advantages like improved mechanical strength and thermal stability, making Nylon 6/MMT a possible ideal alternative for Nylon 66. However, the relationship between the microstructure and physical properties of nylon 6/MMT nanocomposites is unclear so far due to the complicated system, including the highly asymmetric geometry of the exfoliated MMT layer and the complicated interaction between MMT layers and entangled nylon 6 chains. Herein, we focus on two processes, namely the impact of MMT on the solidification procedure during molding and the toughness–brittleness transition during the tensile stretch, in order to elucidate the structure–property relationship of nylon 6/MMT composites. We firstly studied the solidification process of nylon 6/MMT with bending height experiments. The results showed that the solidification process occurs prior to the crystallization of nylon 6, indicating that a physical crosslinked network rather than a crystalline structure is the reason for the solidification process. Furthermore, the solidification speed has a step change at around 2 wt% MMT content, indicating that the MMT percolation network is related to the transition. We further studied the influence of MMT inclusion on the mechanical properties, and found the tensile strain at break showed a similar step change at around 2 wt% MMT content, which further confirms the existence of an MMT percolation network above 2 wt% MMT content. It was generally believed that the main effect of MMT on nylon 6 is the nanofiller enforcement; we found that the percolation effect of the highly asymmetric 2-D nanofiller plays a central role in influencing the mechanical properties and solidification behavior during molding.

## 1. Introduction

Incorporating inorganic nanofillers into a polymer matrix is a facile way to prepare economical and practical polymer-based nanocomposites [1,2,3,4,5], providing the possibility for a large number of applications in industry due to enhanced mechanical stiffness and heat-distortion temperature. Therefore, the research on nanocomposite materials has received extensive attention, which is of great significance for both basic research and industrial application [6,7]. Inorganic montmorillonite nanosheets, as one kind of layered silicates, have attracted much attention due to their easily delaminated layers. Organic modification of MMT makes hydrophilic surface hydrophobic, thus more compatible with a polymer matrix. Compounding organically modified MMT with polymer makes polymer chains enter between MMT layers. Depending on the extent of penetration, the composite structure can be classified into intercalated and exfoliated, where the former shows an increased layer distance from X-ray scattering and the later shows no d-spacing, since the layers are homogeneously distributed in the polymer matrix. Inorganic montmorillonite nanosheets, with a thickness of around 1 nm and size of around 100 nm, is highly asymmetric, making the microstructure and physical properties of nanocomposites more complicated. 

A number of polymers have been used as matrix materials to manufacture polymer/MMT nanocomposites, including polyolefin [8,9,10], epoxy resin [11,12], polyurethane [13,14], polyamide [15] and so on. The research on nylon/montmorillonite nanocomposite materials is a milestone in the research history of nanocomposites, which has led to a new era of polymer-based nanocomposites [16,17,18,19,20]. The operating temperature range of nanocomposite materials is greatly increased because of the enhanced thermal deformation temperature by the exfoliated nano-platelets. Moreover, the gas-liquid barrier property of the polymer base can be effectively enhanced, which makes the application of nylon film greatly expanded due to the lamellar barrier effect [21]. Therefore, the nylon 6/montmorillonite nanocomposites have wide application in film, packaging, structural materials and other fields [22,23,24,25,26].

Since Okada first reported nylon 6/montmorillonite nanocomposites in the 1980s [15,27], the research on nylon 6/montmorillonite nanocomposites has been reported extensively in the past 30 years. However, there are still controversies and deficiencies in the understanding of its structure and properties. In terms of how MMT influences crystallization of nylon 6, there are different arguments. A large number of researchers believe that the montmorillonite layer dispersed in a nylon matrix can be used as a nucleating agent to increase the crystallization temperature of nylon, thus, more importantly, shortening the molding cycle of composite materials [28,29,30]. However, it has also been reported that the addition of montmorillonite reduces the crystallization rate of nylon 6 since montmorillonite reduces the mobility of the chain segment and montmorillonite restricts the growth of the crystal front [31,32]. Besides the influence of MMT on nucleating and crystallization kinetics of nylon 6, Lincoln et al. reported that the composites show an MMT long-range order with a distance of 40 to 60 nm, which disrupts crystalline formation and reduces the crystalline ordering [29]. Lincoln et al. further reported that from SAXS, it is unclear how the addition of silicate sheets influences the long-range lamellar order due to the complexed scattering contribution of fluctuations in polymer melt, dispersion of silicate layers and crystalline order [30]. However, it is still unclear how MMT nanosheets are distributed in the nylon 6 matrix at microscale and correspondingly how the microscale structure influences the final mechanical properties.

In this work, we incorporated organically modified montmorillonite into nylon 6 with a twin screw extruder, and found the interface coupling agents provide good compatibility between MMT nanolayers and the nylon 6 matrix. We studied the solidification process of nylon 6/MMT together with mechanical properties, and found the formation of the percolation network plays a crucial role in influencing the mechanical properties and solidification process during molding. Moreover, the influence of the montmorillonite network structure on the solidification process during molding of materials has been systematically studied and compared with crystallization behavior. The reason for the accelerated solidification of the nylon 6/clay nanocomposite has been proposed to be a physical crosslinked network of MMT. Such a percolation network structure is consistent with the analysis of MMT-influenced mechanical properties, including tensile strain at break and impact energy.

## 2. Experimental part

### 2.1. Materials

Nylon 6 (PA6) used in this experiment was purchased from Kuibyshevatot (Togliatti, Russia, tradename: Volgamid 24). The relative viscosity is 2.4, corresponding to a molecular weight of 16,000 g/mol. Montmorillonite (MMT) treated with organic ammonium(Sinopharm Chemical Reagent Co., Ltd., Shanghai, China), including the carboxyl group (30 wt%), was produced by Zhejiang Fenghong New Material Co., Ltd. (Anji, China, tradename: DK5). Each MMT layer has a thickness of around 1 nm and size of around 100 nm.

### 2.2. Sample preparation

Nylon 6 and montmorillonite were dried at 100 °C and 80 °C for more than 12 h before extrusion. The moisture content of nylon 6 should be controlled below 0.1%. All nanocomposites were prepared in a twin-screw extruder (Haake Process 11 The samples used to measure the bending height was obtained from injection molding by a mini-jet injection at 245 °C followed by a mold at a temperature of 80 °C.

### 2.3. Characterizations

Differential scanning calorimetry. The crystallization and melting behaviors of nylon 6/montmorillonite nanocomposites were analyzed by a differential scanning calorimeter (DSC, Q2000, TA Instruments, New Castle, DE, USA). The sample was heated from 30 °C to 300 °C at a temperature rate of 10 °C/min in the N_2_ atmosphere, then at a constant temperature of 300 °C for 5 min, followed by cooling to 30 °C at the same rate; finally, the second heating is performed under the same conditions as the first heating.

X-ray Diffraction. The crystalline forms of nylon 6/montmorillonite nanocomposites were analyzed with an X-ray diffractometer (Bruker D8, Karlsruhe, Germany). The angle scanning of composites ranges from 2° to 30° at a scanning speed of 2 °C/min.

Electron microscopy. The dispersion of montmorillonite in the matrix was analyzed with a transmission electron microscope (Hitachi HT-7700, Tokyo, Japan). The samples were cut into slices of 90 nm by an ultrathin slicer (Leica EM UC7, Germany) at −50 °C. The samples were observed by transmission electron microscopy after drying. 

Dynamic thermomechanical analysis. The thermal properties of nylon 6/montmorillonite nanocomposites were analyzed with a dynamic mechanical spectrometer (TA Q800, New Castle, DE, USA). The test spline was heated from 0 °C to 230 °C at a temperature rate of 3 °C/min by using the multi-frequency strain mode with a vibration frequency of 5 Hz.

Rheological test. The rheological properties of nylon 6/montmorillonite nanocomposites were analyzed with a rheometer (TA ARES G2, New Castle, DE, USA). The frequency sweep of composites ranges from 0.1 rad/s to 100 rad/s at 235 °C with a fixed strain of 1%. Otherwise, the temperature sweep of composites ranges from 250 °C to 190 °C.

Mechanical test. The tensile stretch experiments were performed on an Instron universal testing machine (model 5966, High Wycombe, UK) at the tensile rate of 10 mm/min Charpy impact tests were carried out on an Impact tester (SS-3700, Sungshu Testing Instrument, Taipei, China) following the GB/T 1843 standard. The specimens were prepared with injection molding with the size of 80.0 × 10.0 × 4.0 mm^3^. Each test was done five times and the average value was used for analysis.

## 3. Results

**Figure 1:**
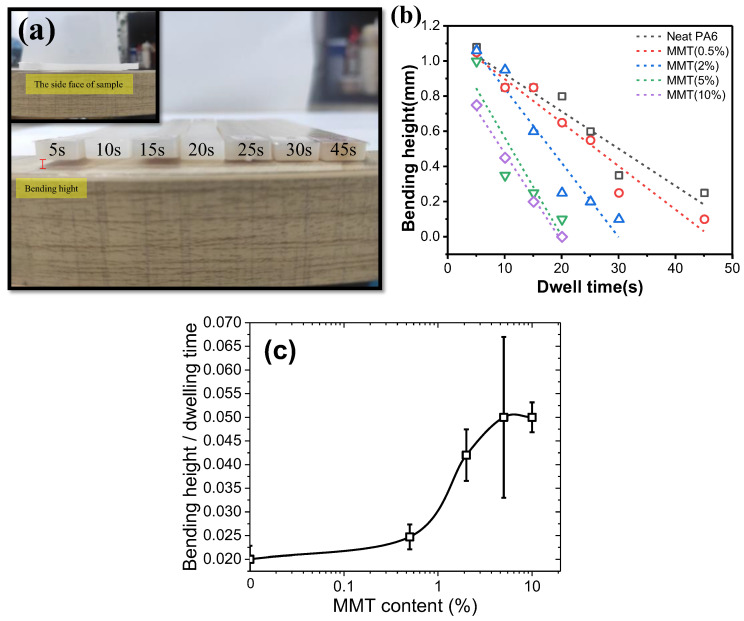
(**a**) Images of neat nylon 6 sample during bending height experiments; (**b**) Bending height as functions of molding dwelling time of nylon 6 and nylon 6/montmorillonite composites with different montmorillonite loadings; (**c**) The ratio of bending height and dwelling time vs. MMT contents.

The industrial products were prepared with injection molding first at 245 °C by a mini-jet and then molded at 80 °C with different dwelling times. Reducing the dwelling time is an efficient way to save production cost. Generally, the dwelling time is strongly dependent on the solidification speed of nanocomposites. The solidification extent can be related to the bending height. The absolute value of bending height vs. dwelling time can be used to describe solidification speed. As shown in Figure 1a, the bending height of neat nylon 6 with dwelling time of 5 s is 1.08 mm, indicating that neat nylon 6 has a significant deformation at shorter dwelling time due to lower modulus. The bending heights were 0.8, 0.35 and 0.25 mm when the dwelling time increased to 20, 30 and 45 s, respectively, indicating a longer molding time facilitated the solidification of nylon 6 and an increased modulus. With increasing MMT content, it was found that the bending height of nanocomposites at the same dwelling time decreases continuously, as shown in Figure 1b. The molding time was shortened to 20 s to reach a bending height of 0.1 mm for nanocomposites containing 5 wt% MMT, allowing for a decent reduction in the production cost. It should be noted that the nanocomposites with 10 wt% MMT have very similar solidification behaviors to those with 5 wt% MMT. 

Here we take the curve slope of bending height vs. dwelling time to represent the solidification speed of nylon 6/MMT composites. As shown in Figure 1c, with increasing MMT content, the solidification speed increases slowly with MMT content, and then a step change occurs at around 2 wt% of MMT content and levels off at around 10 wt% of MMT contents. Overall, the addition of MMT into nylon 6 speeds up the solidification process and the lowering the molding cycle, which increases the molding efficiency and benefits the economical process. However, it is unclear why this happens and why there is such a step change of solidification speed at a MMT composition of 2 wt%. 

A direct influence of MMT on nylon 6 is that such a nanosheet could be used as a nucleation agent, as reported from other researchers although there are also opposing viewpoints that MMT could suppress the crystallization of nylon 6 [30]. Naturally, we want to first verify whether MMT has a nucleation effect so as to improve crystallization of nylon 6 and whether such effect has any relationship to the increased solidification speed.

**Figure 2:**
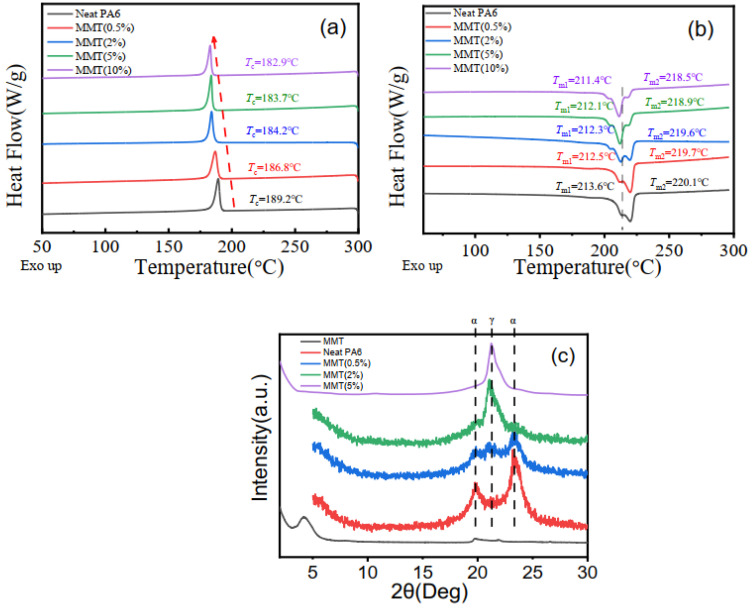
(**a**) DSC cooling curves (the red dashed line to show the trend of peak shift) and (**b**) heating curves of neat nylon 6 and nylon 6/montmorillonite composites with different montmorillonite loadings. The grey dashed line is used to guide the trend of peak shift (**c**) XRD patterns of neat nylon 6 and the nylon 6/montmorillonite composites.

To clarify the origin of enhanced solidification process, thermal analysis was performed to characterize crystallization and melting behavior of the nanocomposites. The DSC cooling scans of neat nylon 6 and nylon 6/montmorillonite composites with different montmorillonite loadings are shown in the Figure 2a. Neat nylon 6 is a semi-crystalline polymer with crystallization temperature of about 189.2 °C and crystallinity of about 33%. The relatively narrow half peak width indicates that nylon 6 has certain crystallization ability even without a nucleating agent because of a strong hydrogen bond among nylon 6 chains. However, incorporating montmorillonite (0.5 wt%) into material leads to a decreased crystallization temperature, indicating a suppressed crystallization behavior. As we can see in the Figure 2a, compared with neat nylon 6, the crystallization temperature of the material with 0.5 wt% montmorillonite drops to 186.8 °C, which is approximately 2.4 °C lower than the values for the neat nylon 6. Meanwhile, the crystallinity of nanocomposite also dropped to 26%. In addition, with increasing montmorillonite content, the crystallization temperature and crystallinity of material decrease continuously. When the inclusion amount of the montmorillonite increases to 2 wt%, 5 wt% and 10 wt%, the crystallization temperature decreases to 184.2 °C, 183.7 °C and 182.9 °C, respectively. The decreased T_c_ and crystallinity suggested a suppressed chain mobility due to the addition of MMT, originating from an interaction between the polar nylon 6 molecular chain and the layer surface of MMT. It should be noted that the crystallization ability of material drops dramatically when montmorillonite is less than 2 wt%, and the decline is slowed down when more montmorillonite was added, as shown in Figure 2b. This phenomenon is related to the structure of the montmorillonite network at a 2 wt% weight fraction. Detailed discussion will be provided in the next section.

Figure 2b shows the melting curves of the montmorillonite/nylon 6 blends. The composites have two endothermic peaks. The lower temperature corresponds to the melting of crystals in γ form, while the higher corresponds to the melting of α form. The addition of montmorillonite (2 wt%) reduces both melting temperatures simultaneously; the higher temperature decreases from 220.1 °C to 219.6 °C, and the lower temperature decreases from 213.6 °C to 212.3 °C. When the additional amount of montmorillonite is 0.5 wt%, 5 wt% and 10 wt%, the higher melting temperatures is 219.7 °C, 218.9 °C and 218.5 °C, and the lower melting temperatures are 212.5 °C, 212.1 °C and 211.4 °C, respectively. With increasing montmorillonite loading, the melting temperature slightly decreases, revealing that adding MMT is unfavorable to the crystallization of nylon 6. Furthermore, it should be noticed that the ordering of γ crystal decreases with MMT loading, as demonstrated with the decreasing melting temperature T_m1_ and the content of less ordered γ crystal increasing with MMT loading, as can be seen from the peak areas corresponding to T_m1_ and T_m2_ respectively. The sample with 10 wt% montmorillonite shows dominating γ crystals with the lowest melting temperature compared to the other MMT compositions. Summarizing the cooling and melting curves of the material, rather than the nucleation effect and improving crystallization of nylon 6, montmorillonite hindered the crystallization process of nylon 6. This might have something to do with the chain mobility of nylon 6, which was greatly suppressed due to strong physical interaction with montmorillonite. 

X-ray diffraction was performed to further understand the effect of MMT on the crystal structure of nylon 6/MMT nanocomposites. Figure 2c shows XRD curves of nylon 6/MMT composites with different MMT loadings. The characteristic reflection peak of montmorillonite appeared at 2θ = 4.2°, corresponding to the typical lamellar structure of MMT layers with basal spacing of 2.08 nm. It needs to be added that the basal spacing of organic modification montmorillonite layer is wider than the natural montmorillonite due to the intercalation agent. When the montmorillonite/nylon 6 blends with different MMT content, the reflection peak of montmorillonite disappears after extrusion. This means that a fully exfoliated nanostructure has been achieved, owing to the nylon chains’ intercalation into the gap of clay platelets. The addition of montmorillonite into nylon 6 matrix does induce great change in the structure of montmorillonite, indicating good compatibility between montmorillonite and nylon chains. As we can see from Figure 2c, incorporation of montmorillonite into nylon 6 leads to a drastic crystal form change compared with the raw nylon 6. Neat nylon 6 exhibits a reflection peak at 20° and 24°, corresponding to the α form crystal, which is a stable crystal structure. The reflection peak of γ form crystal at 21.8° began to appear with a small amount of additives (0.5 wt%), and the reflection peak of γ crystals increased obviously in the montmorillonite(2 wt%)/nylon 6 composite, indicating that the composition of γ form crystal improved substantially, consistent with DSC results above. 

In summary, we can see inclusion of MMT into nylon 6 suppressed the crystallization process of nylon 6. With an increase in MMT content, calorimetry results show that γ crystal forms in place of alpha crystal, and the overall crystallinity changes little, which has also been reported in other literature. XRD results show similar results. It seems that the inclusion of MMT suppressed crystallization instead of enhancing crystallization process, which indicates that solidification speedup of nylon 6/MMT composites was not related to crystallization process.

**Figure 3:**
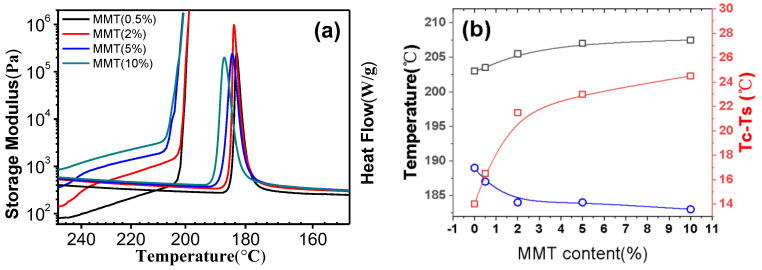
(**a**) The storage modulus and calorimetry results of nylon 6/montmorillonite composites with different montmorillonite loadings during cooling. (**b**) Solidification temperature(red symbols), crystallization temperature(black symbols) and their difference(blue symbols) of neat nylon 6 and nylon 6/MMT composites with different MMT content.

Another way to describe the solidification process is using modulus in rheological experiments. We performed small amplitude oscillatory shear experiments with an angular frequency of 10Hz at a cooling rate of 2 °C/min from 250 °C to 190 °C. Figure 3 shows the storage modulus of nylon 6/MMT composites with varying MMT content against temperature with an angular frequency of 10Hz. A sharp increment in the storage modulus can be observed around 205 °C. Above this temperature range, there is a gradient plateau. Higher MMT content leads to higher modulus, indicating a reinforcement of MMT nanosheets on nylon 6 matrix. Obviously, the temperature corresponding to a sharp G’ increase is higher than the crystallization temperature, meaning the solidification process occurs prior to the crystallization process. As shown in Figure 3b, with increasing MMT content, the difference between the two temperatures increases. All the above results show that before crystallization occurs the solidification process has already happened. Crystallization is not the reason for the solidification process. Note that MMT inclusion didn’t show an obvious influence on glass transition temperature from DSC results (See supplementary materials Figure S1). What could be the reason for the solidification process of nylon 6/MMT nanocomposites? We propose the strong physical interaction between the nylon 6 chain and MMT nanosheets is the reason for the solidification. Studies from the literature also show that MMT has a strong interaction with nylon 6 chains. As shown in Figure 1c, why is there a transition around 2 wt% of MMT content? We propose at around 2 wt% MMT content, the physical crosslinking between MMT and nylon6 chains makes a percolation network of MMT nanosheets. This can be also shown in the literature that a high aspect ratio has a smaller volume fraction for a percolation network [33,34]. Generally, for layered silicates with thickness of 1 nm and length of 100 nm, their critical volume fraction is about 1% [34,35]. The volume fraction of 1% can be converted to a weight fraction of around 2 wt%, close to the transition region of solidification rate as shown in Figure 1c, indicating that such a transition in solidification rate is strongly related to the percolation network formation. Once the MMT volume fraction reaches the percolation threshold, MMT nanosheets acting as crosslinking points have a high enough volume fraction to constitute a physical crosslinked nylon 6 network. The synergistic physical crosslinking causes a sharp increase in the solidification rate of nylon6/MTM nanocomposites. 

**Figure 4:**
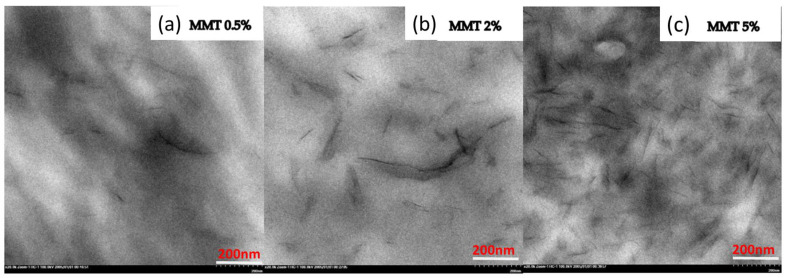
TEM image of nylon 6 with different MMT compositions: (**a**) MMT 0.5%, (**b**) MMT 2%, (**c**) MMT 5%.

Figure 4 shows a TEM image of the nylon 6 with montmorillonite of 0.5 wt%, 2 wt% and 5 wt% loadings. As can be seen from the images, MMT sheets were distributed homogeneously, and no obvious agglomeration of montmorillonite can be observed, demonstrating the exfoliated state of MMT layers and good compatibility with the nylon 6 matrix due to improved interfacial interaction between the nylon chains and MMT sheets. With increasing MMT loading, the number density of MMT increases and the distance between MMT decreases. At a MMT content of 2 wt%, it seems a percolation network forms, while a MMT content of 5 wt% already makes the MMT layers overlapping.

**Figure 5:**
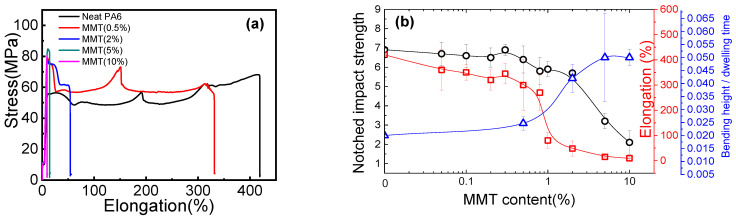
(**a**) Tensile stress strain curves of neat nylon 6 and nylon 6/MMT composites with different MMT composition. (**b**) Notched impact strength, tensile strain at break and bending height/dwelling time of neat nylon 6 and nylon 6/MMT nanocomposites with different MMT composition.

Now we shift to another important property of nylon 6/MMT composites. The literature also shows that with increase MMT content, the tensile strain at break decreases and impact strength energy also decreases [36,37]. We also found similar results, as shown in Figure 5a. The neat nylon 6 shows a tensile strain at break of around 400%. With increasing MMT content, the tensile strain at break of the nanocomposites decreases gradually to 50% at MMT content of 2 wt% and 15% at MMT content of 5 wt%, suggesting a transition from stiffness to brittleness. 

We plot the tensile strain at break and the notched impact strength vs. MMT content, as shown in Figure 5b. We can see that with increasing MMT content, both tensile strain at break and notched impact strength decrease. Given small MMT content, both tensile strain at break and notched impact strength decrease slowly, followed by a dramatic decrease in MMT content of 1 wt% and 4 wt% respectively. Above this MMT content range, both parameters change slowly again. Surprisingly, the solidification speed also shows a similar transition range. It seems that once the percolation network forms, it also influences the mechanical strength strongly. We propose that once the percolation network forms, the polymer matrix is split into small regions, which were mostly connected through MMT layers. Although nylon 6/MMT interaction might be stronger than the nylon 6 chain entanglements, the ductility of the chain is strongly reduced. Energy dissipation during stretching or impact will mostly be counteracted by the detachment of nylon 6 chains from MMT nanosheets. 

This is not uncommon. From the literature [38], we can also see that percolation network formation in polymer/graphene composites, which correspond to conductivity leaping, also coincide with the mechanical transition from stiffness to brittleness. In terms of the mechanical deformation mechanism during nylon 6 tensile stretching, generally there is a first crystal deformation, and then there is chain slippage and crystal orientation during the tensile stretch of nylon 6 [39]. If we check our crosslinking network, once the MMT percolation network forms, it means the continuous entanglement network is somehow blocked by the MMT nanosheets. When this happens, the original long-range chain orientation and slippage were strongly suppressed, replaced by a stress concentration on the nylon 6/MMT interface. Since the physical interaction is strong but lacking deformability and ductility, the composites have a high modulus and a weak tensile strain at break. So the percolation network of MMT nanosheets suppressed the continuous entanglement network, making the impact energy decreases. 

In Table 1, a series of parameters of neat PA6 and composites at different MMT compositions are listed. With increasing MMT loading, Tc, T_d,max_ (TGA results can be found in supplementary materials Figure S2), tensile strain at break and impact energy decrease, while the solidification speed and modulus increase. This means that MMT inclusion suppressed crystallization of nylon 6, while at the same time the thermal stability is also decreased. MMT inclusion increases the elastic modulus while reducing the tensile strain at break. 

What is the relationship between the nylon 6 crystallization and MMT nanosheets? From the modulus measurements during cooling and calorimetry results, we see that the physical crosslinked network of nylon 6/MMT forms is first followed by the crystallization of nylon 6, which means that crystallization of nylon 6 take places under confinement of the MMT percolation network. More MMT nanosheets could be used as nucleation agents of nylon 6 crystals in γ form, which explains the increase in γ crystal forms with an increasing amount of MMT nanosheets. One could imagine that in between MMT nanosheets, nylon 6 crystal lamellae form in between. The closer the lamellae lie with MMT nanosheets, the higher possibility of forming γ crystals, due to the longer relaxation time of large-sized MMT nanosheets and the more retentive orientation of MMT nanosheets after injection molding, as well as that of nylon 6 chains closer to MMT nanosheets. Since at higher MMT contents, nylon 6 matrix was mostly partitioned by MMT nanosheets, most nylon 6 chain ends were attached either on the MMT surfaces via strong physical interactions or lie in between lamellae. Thus, little tie chains exit in between two partitioned areas of nylon 6 chains, causing limited nylon 6 chain orientation and ductile slippage. 

**Figure 6:**
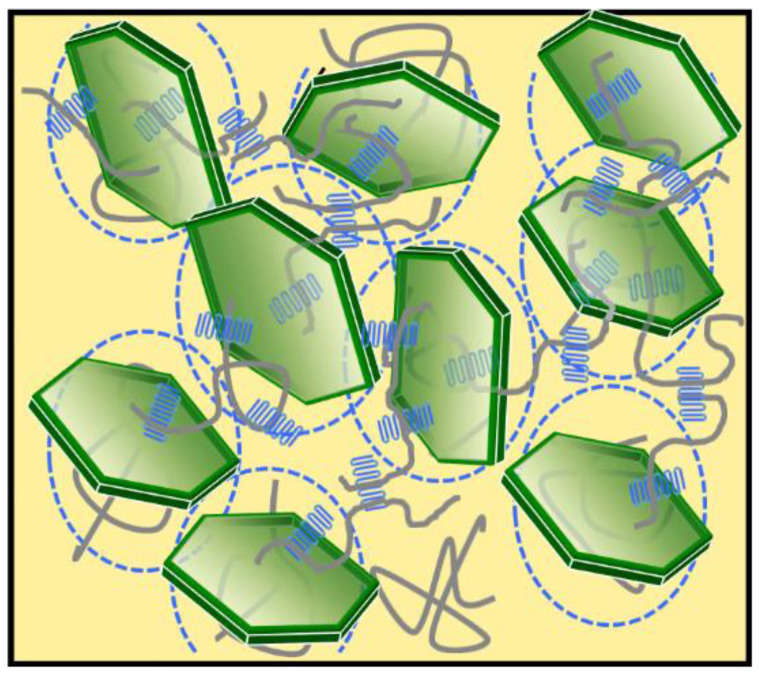
Schematic picture of nylon 6/MMT nanocomposites: a percolation network (physical crosslinked network). Green colored sheets represent MMT nanosheets and the blue dashed line means the effective excluded volume; folded chains in blue are nylon 6 lamellae; Solid line in grey represent nylon 6 chains. Solid grey lines passing through blue lamellar represent part of the chain involved in lamellae.

A schematic picture of the nylon 6/MMT nanocomposites with MMT content higher than 2 wt% is shown in Figure 6. MMT has high enough content so that it forms a percolation network, as shown in the dashed circles in blue. Most nylon 6 chains (shown in grey lines) are confined between MMT nanosheets, which makes the continuous entanglement network isolated and reduces plasticity during stretching. Nylon 6 crystals (shown in blue solid lines) are confined between MMT nanosheets, thus crystallization of nylon 6 was suppressed. Nylon 6 chains could also be involved in different lamellae formation, but chain ends most likely were blocked at MMT surface, which makes bridging chains between lamellae at different regions strongly suppressed. 

## 4. Conclusions

We studied the solidification process of nylon 6/MMT composites, and found inclusion of MMT speeds up the solidification process. Suppression of nylon 6 crystallization with addition of MMT from DSC indicates the solidification process is not related to nylon 6 crystallization. Furthermore, the solidification occurs prior to nylon 6 crystallization, further demonstrating that, rather than nylon 6 crystallization, the physical interaction between MMT and nylon 6 is the reason for nanocomposite solidification. A step change in solidification rate vs. MMT content curve at MMT content of 2 wt% indicates a formation of MMT percolation network. Both tensile strain at break and notch impact energy show a step change vs. MMT content: the composites show a tough property at MMT content below 2 wt%, and a brittleness property above this content. This could be due to the MMT percolation network splitting nylon 6 domains into small parts and penetrating between nylon 6 lamellae, which makes less bridge chains in between nylon 6 crystals lamellae and more nylon 6 chains attached to the MMT layer through physical interaction. Less entanglements lead to suppressed chain ductility. A physical image is given in terms of why higher MMT content leads to mechanical brittleness. 

## Figures and Tables

**Table 1:** List of crystallization temperature Tc, degradation temperature T_d,max_, solidification speed, tensile strain at break and notched impact energy for neat PA6 and composites at different MMT loadings.

	T_c_ (°C)	T_d,max_ (°C)	Solidification Speed (cm/s)	Modulus at 25 °C (MPa)	Tensile Strain at Break (%)	Impact Energy (kJ/m^2^)
Neat PA6	189.2	462.9	0.02	3100	420	6.91
MMT (0.5%)	186.8	465.6	0.0247	4000	300	6.4
MMT (2%)	184.2	465.0	0.042	4200	48	5.7
MMT (5%)	183.7	456.9	0.05	4600	15	3.2
MMT (10%)	182.9	453.6	0.05	4800	9.5	2.1

## Data Availability

The data that support the findings of this study are available from the corresponding author upon reasonable request.

## Supplementary Materials

The following supporting information can be downloaded at: https://www.mdpi.com/article/10.3390/polym14173672/s1: TGA data of nylon 6 and the nanocomposites; DSC curve corresponding to glass transition of nylon 6 and the nanocomposites, Figure S1: Glass transition of PA6 nanocomposites from DSC results; Figure S2: TGA results of neat PA6 and the composites with different MMT loadings.
