# Supplementary material for: Percolation Network Formation in Nylon 6/Montmorillonite Nanocomposites: A Critical Structural Insight and the Impact on Solidification Process and Mechanical Behavior
Source: Polymers (Basel). 2022 Sep 4;14(17):3672. doi: 10.3390/polym14173672 (PMC9460736; doi:10.3390/polym14173672)
Supplement: Supplementary file 1 [file polymers-14-03672-s001.zip › polymers-1882710-supplementary.pdf]

Percolation network formation in Nylon 6/ montmorillonite nanocomposites: a critical structural insight and the impact on solidification process and mechanical behavior

[a] College of Material, Chemistry and Chemical Engineering, Key Laboratory of Organosilicon Chemistry and Material Technology, Ministry of Education, Hangzhou Normal University, Hangzhou, 311121, People's Republic of China

[b] Jiangsu Boiln Plastics Company Limited, Zhangjiagang 215626, People's Republic of China

* Corresponding authors. E-mail address: yongjin-li@hznu.edu.cn (Yongjin Li), tzyan@hznu.edu.cn (Tingzi Yan).

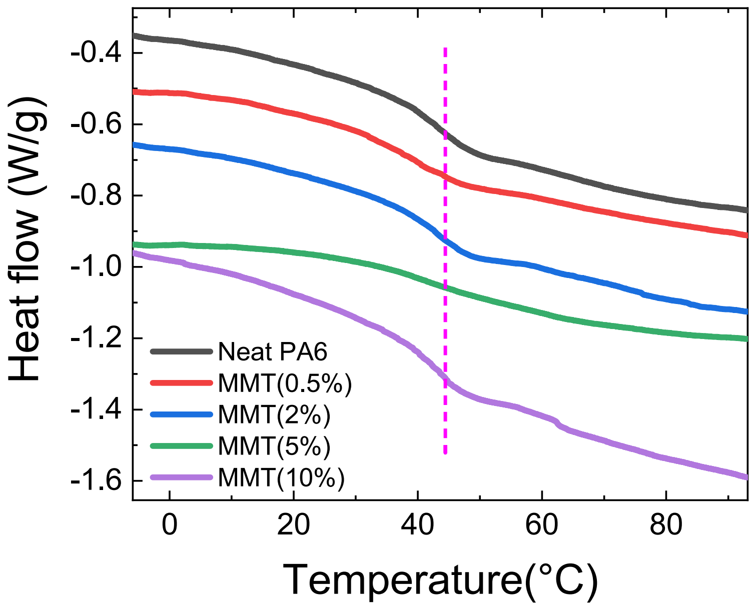

Figure S1. Glass transition of PA6 nanocomposites from DSC results.

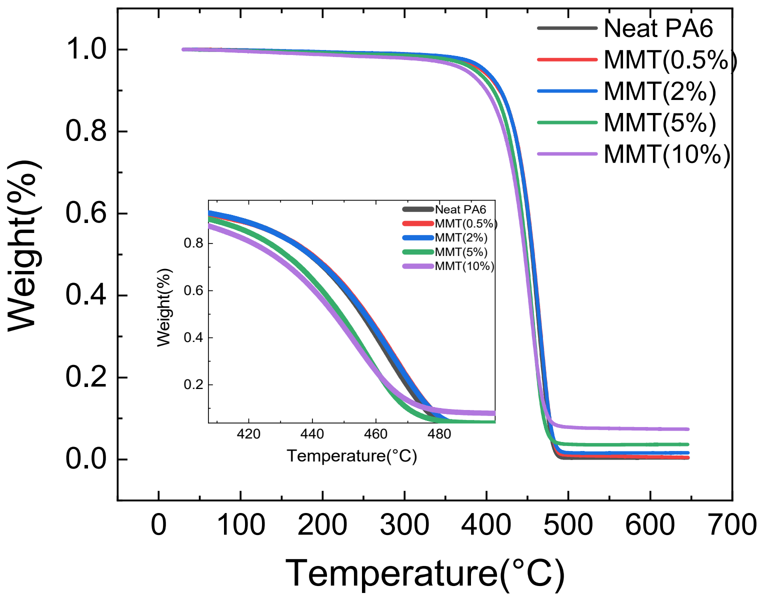

Figure S2: TGA results of neat PA6 and the composites with different MMT loadings.
